# Cholera in Lombardy

**Published:** 1856-01

**Authors:** 


*Cholera in Lombardy.*—The cholera has extended itself over the whole of Italy, and everywhere with destructive ravages. Upper and Middle Italy have chiefly suffered. In Lombardy, from the commencement of the epidemic up to the 19th of August, as to the provinces, and the 25th of August as to the city of Milan, the number affected has been 33,114; of whom 6832 are reported as cured, 15,366 as dead, and 10,916 as under treatment. In Brescia alone, the seizures have been 15,796; of which number the cures are 3596, the deaths 6949, and those remaining under treatment 5251. In the city of Milan the cases have been 295; of whom 40 cured, 182 dead, and 73 under treatment. A writer in the *Annali Universali di Medicina,* attributes the comparative paucity of cases at Milan to the excellent constitution, and admirable preventive measures of the Sanitary Commission of that city. It appears that this body consists of five members, of whom four are physicians. Thus science, directed by experience, guided their deliberations; which were at once productive of appliances, the essential fitness and promptitude of which were neither broken nor retarded by the delays of Bureaucracy. On the contrary, the execution followed instantly on the necessity; and the execution was rigorous, vigilant, and complete, because it was designed to be efficacious. On the other hand, in the provinces it was not the medical men who directed what was requisite for the public health. There, the advice of the physicians, when not utterly rejected, was subjected to non-medical supervision, where the spirit of the prophylactic measures was not adequately comprehended; or, if understood, was ignorantly and inefficiently applied. The results of this are illustrated by several special instances, where medical directions were superseded or thwarted by the magistracy, to the prejudice of the zeal of the practitioners, and the detriment of the public safety.—*Edin. Med. Jour., from Omodei e Calderini, Annali Universali di Medicina, Milano, August* 1855.

